# Complex Scapular Winging following Total Shoulder Arthroplasty in a Patient with Ehlers-Danlos Syndrome

**DOI:** 10.1155/2015/680252

**Published:** 2015-08-12

**Authors:** John G. Skedros, Colton M. Phippen, Tanner D. Langston, Chad S. Mears, Amy L. Trujillo, Robert M. Miska

**Affiliations:** ^1^Department of Orthopaedic Surgery, The University of Utah, Salt Lake City, UT 84108, USA; ^2^Utah Orthopaedic Specialists, Salt Lake City, UT 84107, USA; ^3^Intermountain Medical Center, Salt Lake City, UT 84157, USA; ^4^One to One Physical Therapy, Sandy, UT 84070, USA; ^5^Rocky Mountain Neurological Associates, Salt Lake City, UT 84103, USA; ^6^LDS Hospital, Salt Lake City, UT 84103, USA

## Abstract

This is a unique case of a female patient with features of classical and hypermobile types of Ehlers-Danlos syndrome (EDS) who developed complex scapular winging from spinal accessory and long thoracic neuropathies. These neurological problems became manifest after an uncomplicated total shoulder arthroplasty (TSA). The patient had a complex postoperative course with extensive work-up in addition to revision shoulder surgery and manipulations to treat shoulder stiffness. It was eventually suspected that the periscapular nerve impairments occurred during physical therapy sessions after her TSA. This interpretation was further supported by genetic evidence that, in addition to EDS, the patient had an unrecognized genetic propensity for nerve palsies from stretch or pressure (“hereditary neuropathy with liability to pressure palsies” (HNPP)). By two years after the TSA the neuropathies had only partially improved, leaving the patient with persistent scapular winging and shoulder weakness. With this case we alert surgeons and physical therapists that patients with EDS can have not only a complicated course after TSA, but rare concurrent conditions that can further increase the propensity of neurological injuries that result in compromised shoulder function.

## 1. Introduction

Patients with Ehlers-Danlos syndrome (EDS) typically have hyperextensibility of joints and collagenous tissues [1, 2]. Among the six subtypes of EDS, types I and II are on a continuum that is known as the “classical type” [1]. Type III (hypermobile type) is the most functionally debilitating as well as having the highest frequency of orthopaedic surgeries. Although type III EDS patients have normal wound healing [3], they have high failure rates of soft-tissue procedures when compared to normal patients [4–7]. By contrast, type II EDS is associated with abnormal scar formation [8].

We report a unique case of a female patient with features of classical and hypermobile types of EDS who developed complex scapular winging that resulted from spinal accessory neuropathy and long thoracic neuropathy in the setting of mild brachial plexopathy. These neuropathologies became manifest after what initially seemed to be an uncomplicated total shoulder arthroplasty (TSA).

## 2. Case Report

The patient is a 45-year-old right-hand-dominant female (height: 180 cm; weight: 68 kg; BMI: 21) who presented to our clinic on April 25, 2013, with a chief complaint of moderate-to-severe right shoulder pain. For two years, right arm elevation greater than 80° was not possible because of pain. Magnetic resonance (MR) imaging revealed end-stage arthritic changes of the right glenohumeral arthritis but no rotator cuff tear or significant muscle atrophy. Ten years prior, she had a successful repair of a two-centimeter supraspinatus tendon tear and had eight years of over-head shoulder motion. She worked as a nurse in an outpatient clinic and trained horses as a hobby. For nearly a decade, she was in chronic pain management for myofascial pain syndrome, cervical radiculitis, and polyarthralgia (these were her diagnoses before the diagnosis of EDS was established). Three years prior, and based on clinical criteria, she was diagnosed with EDS (with features of types II and III).

**Figure 1:**
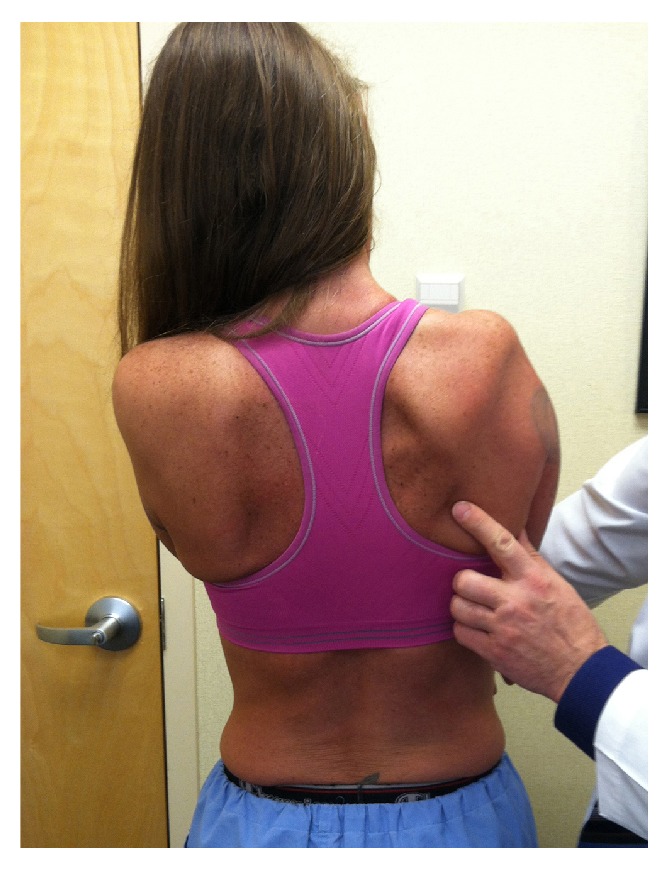
Photo taken at four months after the index TSA shows the patient's right scapular winging with clear atrophy of the trapezius muscle. The patient is pressing her hands against the wall in front of her.

A standard right total shoulder arthroplasty (TSA) was performed on May 7, 2013, by JGS. Before the induction of general endotracheal anesthesia, an interscalene nerve block was done with ultrasound guidance without difficulty [9]. Over the following three months, she complained of increasing weakness and pain in her right shoulder region. Two months after the TSA, mild scapular winging was identified and was felt to be secondary to pain-related compensatory motion of the pectoral girdle [10]. During physical therapy sessions the manual shoulder stretching caused unusually high pain. By 3.5 months after the TSA, the scapular winging was more pronounced and there was obvious atrophy of the ipsilateral trapezius (Figure 1). There was pectoralis major weakness and decreased sensation over the right index finger and lateral forearm. An electromyography (EMG) and nerve conduction study (NCS) revealed (1) impairment of right spinal accessory nerve and the trapezius muscle (mostly the middle and lower portions) with motor unit changes consistent with axonal loss but without ongoing denervation, (2) diffuse motor unit changes in the middle/lower trunk distribution without impairment of the axillary or suprascapular nerves, and (3) axonal loss of the median nerve with evidence of mild reinnervation. The serratus anterior was not impaired. The pectoralis major muscle was not evaluated.

At five months after the TSA, cervical (C) spine MR imaging revealed prominent multilevel neural foraminal narrowing, which was not significantly changed when compared to MR imaging done three years prior. The current findings included mild multiple-level spondylosis that was moderately severe at C6-C7 and moderate at C4-C5 and mild spinal stenosis at C5-C6 and C6-C7. A MR neurogram [11] of the spinal accessory nerve showed no abnormality along the entire nerve or in the brain.

The patient's high level of pain led to expansion of the differential diagnosis to include complex regional pain syndrome (CRPS, type 1) [12] and Parsonage-Turner syndrome (neuralgic amyotrophy) [13–15] with involvement of the spinal accessory nerve [16–20]. However, two consultants (a head and neck surgeon and a neurologist (coauthor RMM)) thought that these diagnoses were unlikely. RMM hypothesized that although EDS likely contributed to her symptoms by enhancing the possibility of tissue stretching during the TSA surgery and/or in subsequent physical therapy, the neuropathologies were not likely solely caused by EDS. Multiple mononeuropathy was then considered an underlying neurological condition, with the possible etiology of “hereditary neuropathy with liability to pressure palsies” (HNPP) in addition to the patient's EDS [21–24]. RMM had seen the patient three years prior. In the current (2013) examination there were additional neurological findings that resembled some of those seen in 2010: anterolateral right thigh numbness, bilateral leg and dorsolateral foot numbness, as well as numbness of the dorsum of each hand, and variable numbness on the volar forearm (especially right) and numbness in the first through third fingers on the palmar surface (worse on left). Genetic testing (Athena Laboratories Inc., Worcester, MA, USA) revealed a variant in the PMP22 gene (technical result: c.320-4C>T) that was consistent with HNPP; the clinical significance of the patient's gene variant was ranked as 4 cm on a 10 cm scale where 0 is “benign” and 10 is “pathogenic.”

**Figure 2:**
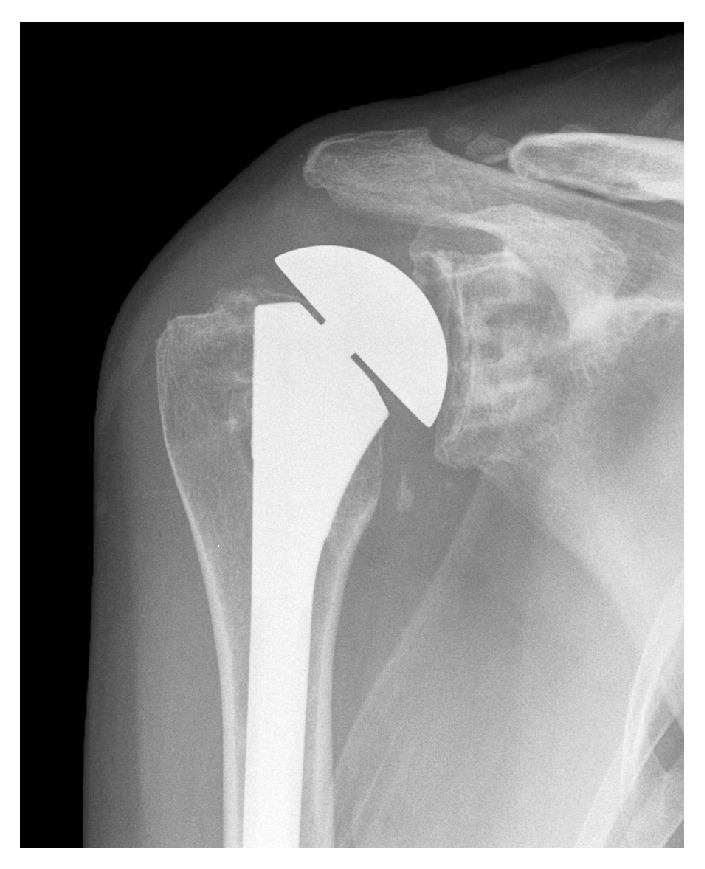
Anterior-posterior radiograph of the patient's right shoulder taken seven months after the index TSA. The distance between the acromion and humeral head is not narrowed in this image. This contrasts with the later radiograph shown in Figure 3 where this distance is significantly narrowed (which suggests weakness of the superior rotator cuff).

At eight months after the index TSA, an independent shoulder surgeon was consulted. A CT arthrogram of the patient's right shoulder revealed no evidence of a rotator cuff tear or prosthesis loosening. Additionally, a fluoroscopic-guided aspiration of the right shoulder revealed no evidence of infection. His opinion was to (1) observe the scapular winging [25–27] and (2) perform revision shoulder surgery to manually lyse adhesions and revise the humeral prosthesis to reduce its overall vertical “height” in order to correct “overstuffing” (Figure 2).

**Figure 3:**
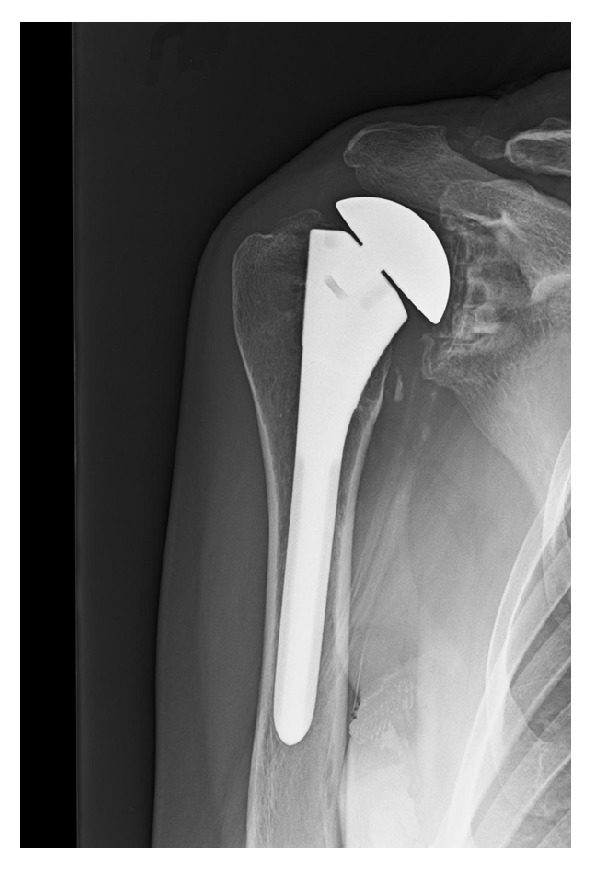
Anterior-posterior radiograph of the patient's right shoulder taken eight months after the revision of the humeral head (16 months after the index TSA). Narrowing of the distance between the acromion and the humeral head seen in this image is suggestive of weakness or a tear of the superior rotator cuff [28].

At nine months after the index TSA, the trapezius palsy had not improved. Revision surgery was performed by the same surgeon (JGS) who performed the index TSA. Intraoperative frozen sections of glenohumeral synovial tissue showed no abnormality. The glenoid component was not loose and the humeral head was replaced with a smaller humeral head. This reduced the height by a sufficient amount to allow functional shoulder motion in patients with normal rotator cuff strength [29–31]. The well-fixed humeral stem was retained (Figure 3). Intra- and extra-articular lysis of adhesions with an anterior capsular Z-lengthening were done in addition to an open right carpal tunnel release.

By one year after the index TSA the patient's trapezius atrophy was only mildly improved. A repeat NCS/EMG (done 13 months after the index TSA) showed (1) serratus anterior recruitment was decreased compared to the previous EMG, (2) the pectoralis major muscle (not examined in the first study) showed motor unit changes consistent with previous axonal loss with reinnervation, and (3) motor units in the upper, middle, and lower portions of the trapezius muscle were present, which was considered an indication of improvement. Consultation with another physical therapist was then obtained because of the patient's continuing complaints of “intolerable pain” during physical therapy sessions, which likely exacerbated the scapular dyskinesia [10, 32]. This is when it became clear that the initial physical therapist had not been manually stabilizing the scapula while forcefully stretching the patient's upper extremity—this likely produced more scapulothoracic motion than glenohumeral motion. We hypothesized that this caused stretch injuries to the spinal accessory and long thoracic nerves [33, 34].

**Figure 4:**
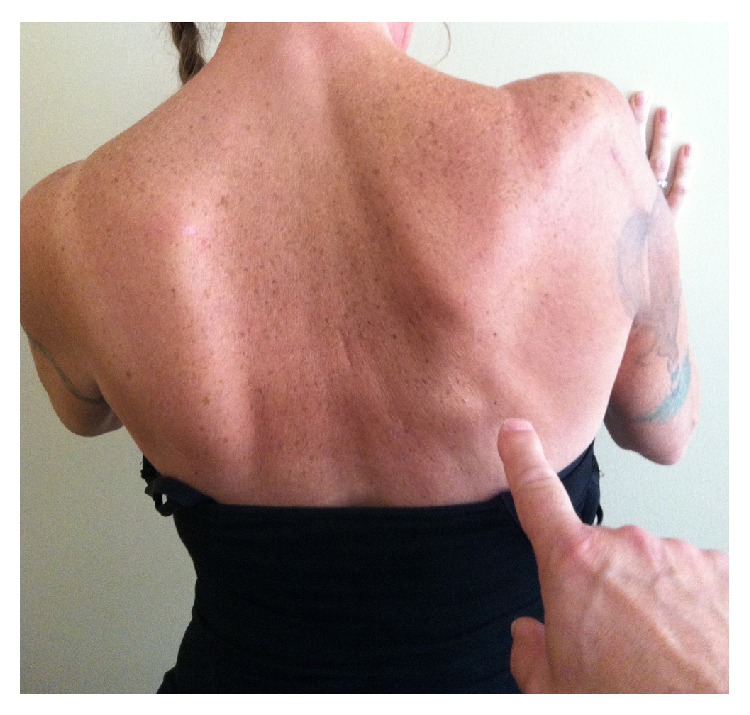
Photo taken 18 months after the index TSA shows the patient's back while she presses her hands against the wall in front of her. Although scapular winging is still obvious, there is increased mass of the trapezius when compared to the image taken 14 months prior (Figure 1). Although periscapular muscle tone had improved, shoulder function was not significantly changed.

A second MUA of the right shoulder was done (July 1, 2014) under general anesthesia in addition to arthroscopic debridement of scar tissue. This procedure was followed by a new therapy regime that significantly increased passive motion, and mild improvements in trapezius tone were continuing, especially of the middle and lower portions. Additional modalities included (1) far infrared light therapy [35, 36] of the scapulothoracic and glenohumeral regions and (2) a neuromuscular stimulator (Empi 300 PV, DJO Global, Vista, CA, USA) [37] for the lower and middle trapezius muscles. However, moderate medial and mild lateral scapular winging continued and active elevation at the shoulder over 90° was not possible in the standing position (Figure 4).

**Figure 5:**
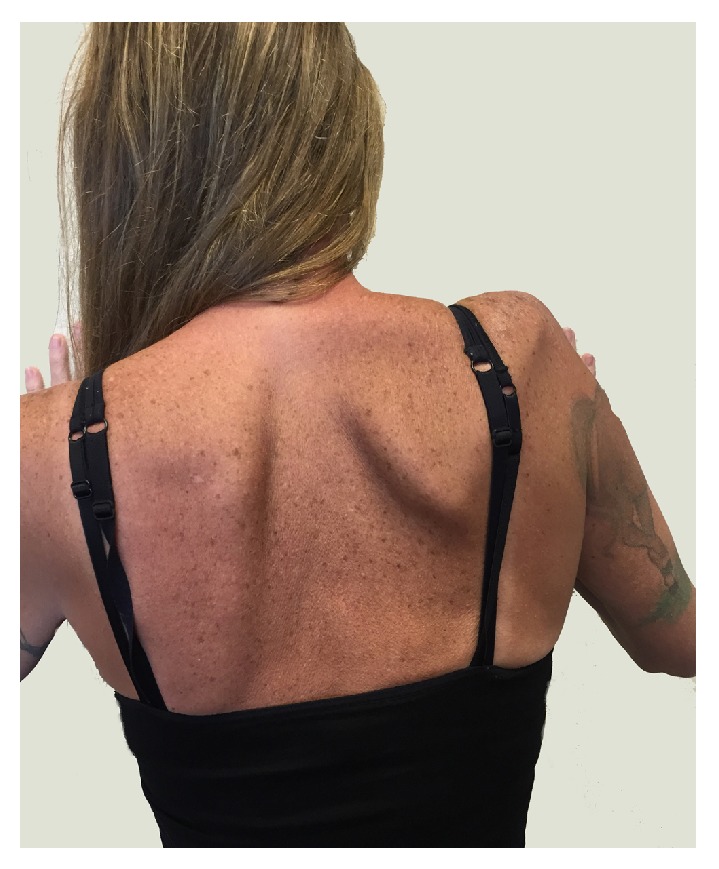
Photo taken 24 months after the index TSA shows the patient's back while she presses her hands against the wall in front of her. The trapezius atrophy has worsened and the lateral scapular winging is more pronounced than six months prior (Figure 4).

The patient was informed that active motion above 90° would be difficult, if not impossible, to achieve without stability of the pectoral girdle during dynamic shoulder motion [38–40]. The plan was to continue to observe the trapezius palsy because of the likelihood that it would spontaneously improve to an acceptable level of strength [25–27]. If the scapular winging did not spontaneously improve by 18 to 24 months after the index TSA, then the plan was to restore the deficient trapezius with a modified Eden-Lange procedure [25, 40]. At 18 months after the index TSA, cervical spine MR scans were obtained with flexion, extension, and neutral positions and showed no significant nerve root or spinal cord impingement (i.e., findings similar to the MRI performed 13 months prior). At two years after the index TSA, the patient's shoulder pain had mildly improved but her shoulder function had not improved when compared to her preoperative status. No additional surgery was planned and the patient was highly unsatisfied with her shoulder function (Figure 5).

## 3. Discussion

We initially speculated that intraoperative patient positioning was the proximate extrinsic cause of our patient's SAN (LTN was recognized later) and that EDS increased the propensity of stretch injury [15, 18]. When the association with surgery was deemed highly unlikely, neurological conditions were considered. Although examples of upper limb neuropathies, as in our patient, have been frequently described in EDS, neuropathies limited to the lower limb in EDS are apparently less common [41], and patients with EDS and upper and lower limb neuropathies are probably rare [42–44]. The coexistence of EDS and HNPP in our patient makes her case even more unusual because we could only find three cases where this has been reported [44–46].

Although spontaneous SAN of unknown origin has been reported [47–52], none of the patients in these reports were known to have EDS. The main extrinsic cause of our patient's periscapular neuropathies may have been the manner of the physical therapy that was performed—similar to stretch injuries reported in therapy and nontherapy circumstances [25, 33, 34, 53–55]. The diagnosis of EDS along with HNPP likely increased the propensity for nerve trauma [12, 18, 21, 22, 43, 46, 56, 57].

The modified Eden-Lange procedure, which can correct lateral scapular winging from trapezius deficiency [40, 58–60], was not performed because (1) spontaneous improvement in trapezius strength was considered likely [26, 27] and (2) the patient's hyperextensible collagenous tissues would increase the probability that this procedure would fail. We initially expected spontaneous improvement because the association of SAN with our patient's index TSA resembled case reports where SAN occurred in association with open-heart surgery [26, 27]. In these cases the SAN was likely caused by pressure/strength injuries from positioning during the surgery or by a complication of internal jugular vein cannulation (this was not done on our patient) [61–63]. These favorable outcomes are consistent with prior studies that have also implied or showed that conservative management can produce acceptable outcomes when SAN or LTN are idiopathic or are caused by stretch injury [25, 40, 53, 64]. But it was not possible to apply the conventional recommendations of when to possibly intervene with muscle-transfer surgery for our patient because none of the patients described in these prior reports had been diagnosed with both SAN and LTN or had characteristics of EDS or HNPP.

In addition to being correlated with type II EDS (where abnormal scar formation can occur), our patient's painful and stiff shoulder after the TSA may have been influenced by “overstuffing” of the glenohumeral joint. As shown in Figure 2, the humeral head of the index TSA was approximately 15 mm prominent (i.e., increased “height” and “offset”) with respect to the location of the surgical neck. But in view of the results of Nyffeler et al. [31] we felt that this was of lesser significance in causing shoulder dysfunction when compared to the scapular winging. Nevertheless, we revised the patient's humeral prosthesis (Figure 3) to reduce the overall height to an amount that several studies have shown to not significantly impair shoulder function in patients with normal strength [29–31]. Our patient's shoulder weakness in elevation continued, which we attributed to intrinsic muscle weakness that occurs in EDS [42, 65] in the setting of her acquired/persistent scapular winging.

In summary, the coexistence of EDS and HNPP is rare, and these conditions contributed to our patient's poor result at two years after a TSA. She felt that the TSA had not significantly improved her shoulder function and only mildly improved her shoulder pain. Surgeons should therefore be cautious of performing this procedure on EDS patients and also be aware that there can be additional neurological conditions that can compromise a good outcome. Surgeons and physical therapists should also recognize that these patients are prone to nerve stretch injuries.
